# Expression, localization and polymorphisms of the nuclear receptor PXR in Barrett's esophagus and esophageal adenocarcinoma

**DOI:** 10.1186/1471-230X-11-108

**Published:** 2011-10-06

**Authors:** Anouk van de Winkel, Vivianda Menke, Astrid Capello, Leon MG Moons, Raymond GJ Pot, Herman van Dekken, Peter D Siersema, Johannes G Kusters, Luc JW van der Laan, Ernst J Kuipers

**Affiliations:** 1 Department of Gastroenterology and Hepatology, Erasmus University Medical Center Rotterdam, Rotterdam, The Netherlands; 2 Department of Pathology, Erasmus University Medical Center Rotterdam, Rotterdam, The Netherlands; 3 Department of Surgery, Erasmus University Medical Center Rotterdam, Rotterdam, The Netherlands; 4 Department of Internal Medicine, Erasmus University Medical Center Rotterdam, Rotterdam, The Netherlands

## Abstract

**Background:**

The continuous exposure of esophageal epithelium to refluxate may induce ectopic expression of bile-responsive genes and contribute to the development of Barrett's esophagus (BE) and esophageal adenocarcinoma. In normal physiology of the gut and liver, the nuclear receptor Pregnane × Receptor (PXR) is an important factor in the detoxification of xenobiotics and bile acid homeostasis. This study aimed to investigate the expression and genetic variation of PXR in reflux esophagitis (RE), Barrett's esophagus (BE) and esophageal adenocarcinoma.

**Methods:**

PXR mRNA levels and protein expression were determined in biopsies from patients with adenocarcinoma, BE, or RE, and healthy controls. Esophageal cell lines were stimulated with lithocholic acid and rifampicin. PXR polymorphisms 25385C/T, 7635A/G, and 8055C/T were genotyped in 249 BE patients, 233 RE patients, and 201 controls matched for age and gender.

**Results:**

PXR mRNA levels were significantly higher in adenocarcinoma tissue and columnar Barrett's epithelium, compared to squamous epithelium of these BE patients (*P *< 0.001), and RE patients (*P *= 0.003). Immunohistochemical staining of PXR showed predominantly cytoplasmic expression in BE tissue, whereas nuclear expression was found in adenocarcinoma tissue. In cell lines, stimulation with lithocholic acid did not increase PXR mRNA levels, but did induce nuclear translocation of PXR protein. Genotyping of the PXR 7635A/G polymorphism revealed that the G allele was significantly more prevalent in BE than in RE or controls (*P *= 0.037).

**Conclusions:**

PXR expresses in BE and adenocarcinoma tissue, and showed nuclear localization in adenocarcinoma tissue. Upon stimulation with lithocholic acid, PXR translocates to the nuclei of OE19 adenocarcinoma cells. Together with the observed association of a PXR polymorphism and BE, this data implies that PXR may have a function in prediction and treatment of esophageal disease.

## Background

Persistent regurgitation of gastroduodenal contents into the lower esophagus causes mucosal injury manifested as reflux esophagitis (RE) [1,2]. As a complication of chronic RE, a Barrett's esophagus (BE) can develop [3,4]. BE is defined as an acquired condition in which the stratified squamous epithelium of the lower esophagus is replaced by specialized intestinal epithelium [5]. It is the sole commonly recognized risk factor for the development of esophageal adenocarcinoma (EAC) [6,7] and has an increasing incidence in the Western world [8]. While the importance of acid and bile exposure in the development of BE is well established [1,5,9,10], only a small percentage of BE patients will ultimately develop EAC. It remains largely unclear which factors control the rate of neoplastic progression in BE [11]. A growing body of evidence suggests that the intrinsic adaptive response to the toxic bile acids from the gastroduodenal contents is unable to prevent injury to the esophageal lining, thus suggesting a role for bile-induced signalling in the progression of BE [12].

An important step in understanding the adaptive defence mechanism against toxic substances has been the identification and characterization of the nuclear pregnane × receptor (PXR) [13-16]. PXR belongs to the nuclear receptor subfamily of ligand-activated transcription factors that play a key role in the regulation of biliary transport systems and enzymes that confer a protective role against toxic bile acids [12]. This group of nuclear receptors includes the constitutive androstrane receptor and the vitamin D receptor [17,18]. In humans, PXR is most abundantly found in the liver, the small intestine and the colon [13,15,16,19]. It is activated by a structurally diverse array of xenobiotics and endogenous compounds, including bile acids and steroid hormones [13,17,18]. Variability at the PXR genetic locus is therefore thought to be associated with pathophysiological changes in steroid, cholesterol or bile acid levels [14]. Polymorphisms in the PXR gene are associated with diseases such as inflammatory bowel disease and primary sclerosing cholangitis [20,21]. As these chronic inflammatory diseases are associated with aberrant bile acid metabolism, there may also be a link between PXR and BE.

The specific aim of this study was to explore the expression and distribution of PXR in BE and adenocarcinoma patients and analyse possible associations in the PXR gene with esophageal disease. We show that PXR expresses in tissue of BE and adenocarcinoma patients, and that it translocates to the nucleus in esophageal adenocarcinoma cells upon bile acid stimulation. In addition, a link between PXR polymorphisms and esophageal disease was found.

## Methods

### Human specimens

For immunohistochemistry, multiple biopsies of adenocarcinoma tissue (n = 19), columnar epithelium from BE patients without dysplsia (n = 28) and squamous epithelium from RE patients (n = 8) were taken at the same distance from the z-line. As healthy controls we included subjects that had no gastroesophageal reflux disease (GERD) symptoms or endoscopically detected aberrations of the esophagus (n = 3). The number of biopsies taken was approximately four per patient, and varied between one and eight biopsies. For each patient, all biopsy specimens were embedded in one single block of paraffin and were therefore stained and analyzed in one slide. Histologic diagnosis was made by two experienced gastrointestinal pathologists (HD and HV). All patients had specialized intestinal metaplasia and were graded according to the most severe stage found. Cases on which agreement could not be reached or that were indefinite for dysplasia were excluded from this study.

Table 1 gives patient characteristics of the population used for analysis of PXR mRNA levels. mRNA levels were determined in a total of 119 esophageal samples, counting biopsies from 11 adenocarcinoma patients, duplicate biopsies of both the squamous and the columnar epithelium from BE patients (n = 21), squamous epithelium of RE patients (n = 7), and squamous epithelium of healthy controls (n = 5) without GERD symptoms or endoscopically detected aberrations of the esophagus. All BE patients had histologically confirmed intestinal metaplasia without high-grade dysplasia.

**Table 1:** Patient characteristics for PXR mRNA analysis

	RE (n = 7)	BE (n = 21)	EAC (n = 11)
Age, y (range)*	43 (21-60)	61 (34-78)	62 (42-73)
Male, (%)*	71	71	82
Type of epithelium	Sq	Sq, CE	tumor

RE: reflux esophagitis, BE: Barrett's esophagus, EAC: esophageal adenocarcinoma, Sq: squamous epithelium, CE: columnar epithelium

*Groups did not differ significantly in gender. As expected, BE and EAC patients were somewhat older than RE patients

Characteristics of the group included in this study for genotyping are shown in Table 2. The total of 683 genetically unrelated Caucasians included 249 BE patients, 233 RE patients and 201 controls without any history of GERD symptoms, who all visited the endoscopy unit of the Erasmus MC-University Medical Center Rotterdam or the IJsselland Hospital in Capelle aan den IJssel between November 2002 and February 2005 [22]. This study was approved by the institutional ethics review committees, and all patients gave informed consent before participating in the study.

**Table 2:** Patient characteristics per group for genotyping

	HC (n = 201)	RE (n = 233)	BE (n = 249)
Age, y (range)	57 (18-90)	54 (19-88)	61 (33-95)
Male, (%)	57	54	69
Length of BE segment, cm (SD)	NA	0	4.23 (2.39)

HC: healthy controls, RE: reflux esophagitis, BE: Barrett's esophagus, NA: not applicable

### Cell lines

The human adenocarcinoma cell line OE19 and human squamous epithelial cell line HET1A were obtained from the ATCC. OE19 cells were grown in RPMI 1640 supplemented with 10% fetal calf serum (FCS), 2 mmol/l glutamine, 100 units/ml penicilline and streptomycin. HET1A cells were cultured in serum-free BRFF-EPM2 medium supplemented with 100 units/ml penicilline and streptomycin. Cells were maintained routinely at 37°C in 5% CO_2 _humidified atmosphere. After a period of at least 24 h to allow cells to adhere they were stimulated with 10 μM of rifampicine, 50 μM lithocholic acid (LCA), or 50 or 100 μM taurolithocholic acid (TLCA) for 24 h.

### Real-Time PCR mRNA quantification from human esophagus samples

Total RNA was extracted from tissue biopsies using TriReagent (Sigma, St Louis, MO) and purified using an RNeasy micro column kit (Qiagen, Hilden, CA). One-fortieth of a 1 μg cDNA synthesis reaction (iScript cDNA Synthesis Kit; Bio-Rad) was used in a 25 μl Real Time-PCR using SYBR GreenER (Invitrogen, Carlsbad, CA). The following primers were used for PXR gene amplification: 5'- ATGGCAGTGTCTGGAACTAC-3' and 5'- CAGTTGACACAGCTCGAAAG-3'. Duplicate samples were run three times in independent PCR runs and the average level of PXR was normalized to GAPDH using the ΔCt method [23].

### Immunohistochemistry

Formalin fixed, paraffin embedded, five μm sections were mouned on glass slides. After deparaffinization in xylene and dehydration in alcohol, endogenous peroxidase was inactivated by incubation with 1% hydrogen peroxidase in methanol for 20 min. Microwave pretreatment in glycin-HCl/EDTA buffer (50 mM Glycin, 10 mM EDTA, pH 3.5) was performed for 10 min. After treatment with 10% normal human plasma/10% goat serum to block non-specific antibody binding, sections were incubated overnight at 4°C with a rabbit anti-human PXR antibody (diluted 1:200, clone poly6169; Biolegend; San Diego, USA), followed by a biotin-labeled mouse anti-rabbit IgG (diluted 1:200; Dako, Glostrup, Danmark), and streptavidin-horseradish peroxidase (diluted 1:300, Dako) and visualized with diaminobenzidine. Nonspecific background controls were done by omitting the primary antibody and an isotype control was included. Samples of the terminal ileum served as a positive control. Sections were evaluated at a 200- and 400-fold magnification using light microscopy (Axioskop 20, Zeiss) by two independent observers (AW and KZ). At least 100 cells were counted in representative areas of longitudinally sectioned crypts in BE cases or high power fields in adenocarcinoma cases. For quantification only cases with nuclear protein expression were considered PXR positive, with cases evaluated as positive for PXR when more than 2% of counted cells showed nuclear positivity of PXR protein.

### Confocal microscopy

Cells were cultured on coverslips washed with phosphate buffered saline (PBS) and fixed with 2% paraformaldehyde for 10 min. After washing, cells were permeabilized with 0.2% Triton ×100 for 20 min and then blocked with 5% goat serum and 5% normal human plasma in PBS with 5% BSA. Cells were incubated with mouse IgG or anti-hPXR antibody (1:200; Biolegend, San Diego, USA) at 4°C overnight and then probed with 1:200 dilution of goat anti-rabbit Alexafluor 594 (Invitrogen; Oregon, USA). Hoechst 33342 was used to stain nuclei. Coverslips were mounted onto glass slides with gelvatol and visualized under a Zeiss LSM 410 laser-scanning confocal microscope (Zeiss, Oberkochen, Germany).

### Genotyping

Genomic DNA was extracted from 5 ml of whole blood by a wizard genomic DNA purification kit (Promega, Madison, USA). We analyzed polymorphisms -25385C/T, 7635A/G, and 8055C/T as these should be informative for eight PXR polymorphisms and were observed by Zhang *et al *[14] to have an effect on PXR function in humans. Assay validation setup was performed by K-Biosciences (Herts, UK) before performing a double blind analysis of PXR SNPs with a competitive allele-specific PCR system using primers designed in flanking region of the SNP located at -25385; TGGTCATTTTTTGGCAATCCCAGGTT[C/T]TCTTTTCTAC CTGTTTGCTCAATCG at 7635; AGGAGCCATCCTCCCTCTTCCTCTC[A/G]CCCCCAA CTTCTGGATTATGGGATG and at 8055; GCTTGCTGAGAAGCTGCCCCTCCAT[C/T]CT GTTACCATCCACAGGTGGCTTCC of the PXR gene NR1I2.

### Statistical analyses

The study was powered (80%) to allow detection of a 10% difference in genotype distribution of the PXR polymorphisms between the groups by performing Chi-square analysis. Odds ratio (OR) and 95% confidence interval (95% CI) were calculated by risk estimate analysis. All statistical analyses were conducted using SPSS v11.0 (SPSS, Chicago, IL) and two-sided significance was taken as *P *< 0.05.

## Results

### PXR gene expression is elevated in BE and adenocarcinoma

**Figure 1:**
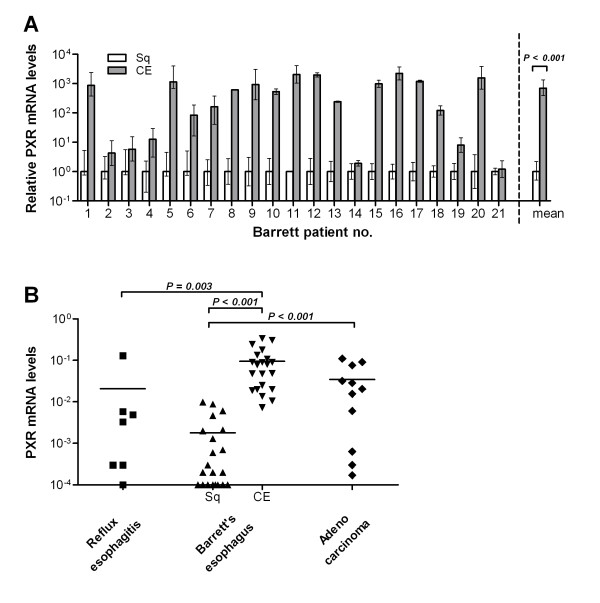
**Relative mRNA levels of PXR in esophageal epithelium as determined by quantitative Real-Time PCR**. (A) PXR levels in columnar epithelium (CE) are normalized to adjacent squamous epithelium (Sq) using 2^(-ΔΔCt) ^method [23] and are plotted for each of 21 Barrett's esophagus (BE) patients. Error bars express a range which is a result of incorporating the standard deviation into the calculation. The mean of this population renders a strong significant increase of PXR mRNA levels in CE compared to Sq of BE patients (*P *< 0.001). (B) PXR mRNA levels are calculated using 2^(-ΔCt) ^to show interindividual differences in PXR expression in RE, BE, and EAC patients and plotted on a log scale. Levels in Sq from patients with reflux esophagitis, and patients with BE are lower than in CE derived from the esophagus of BE patients (*P *= 0.003 and *P *< 0.001 respectively). mRNA levels in tissue from adenocarcinoma patients did not differ statistically from CE of BE patients, but was significantly higher than all Sq tissues. The detection limit for this assay was 0.0001.

PXR mRNA was determined by Real-Time PCR in a group of 44 subjects with different esophageal pathologies (Table 1). As shown in Figure 1A, levels of PXR mRNA were found consistently higher in columnar tissue compared to matching squamous tissue (*P *< 0.001), in which levels of PXR transcripts were barely detectable. Figure 1B shows interindividual differences in PXR expression between RE, squamous and columnar epithelium of BE, and EAC. The levels of PXR mRNA in the BE columnar epithelium were higher than in squamous epithelium of RE (*P *= 0.003, Figure 1B) and healthy controls (*P *= 0.002, data not shown). Also PXR gene expression in tissue of adenocarcinoma patients was significantly higher than in squamous samples from BE patients and healthy controls. Comparing RE with controls, only one patient showed a strong increase in PXR mRNA and thus overall difference in mRNA levels between these two groups did not reach statistic significance (Figure 1B).

### PXR protein distribution in BE and adenocarcinoma tissue

**Figure 2:**
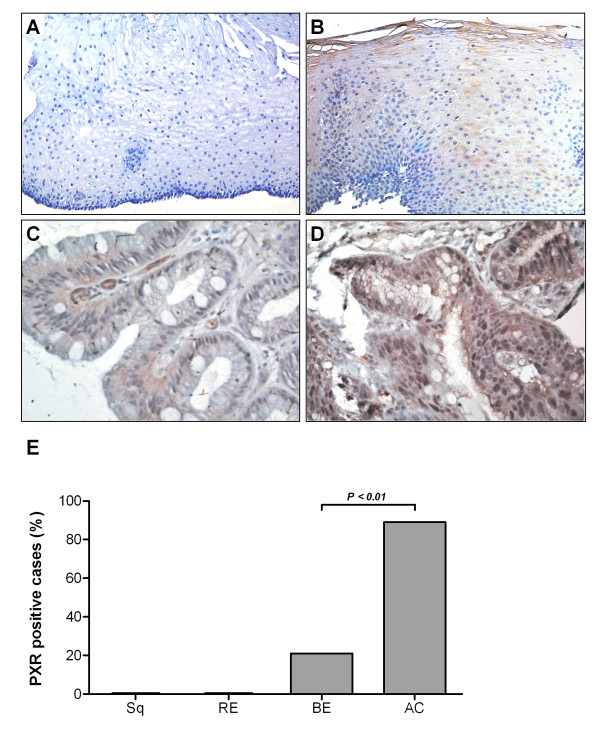
**Result of PXR immunohistochemical staining on esophageal biopsy specimens**. (A) Esophagus of healthy controls is lined by a stratified squamous epithelium and is negative for PXR (200×). (B) The esophageal mucosa of patients with reflux esophagitis is damaged and inflamed and demonstrates a weak signal for PXR (brown) in the cytoplasm of epithelium (200×). (C) Barrett's epithelium is characterized by a single layer of columnar epithelium with the presence of typical goblet cells. Cytoplasmic as well as some nuclear PXR expression is observed (400×). (D) Cells of adenoma tissue show high levels of nuclear PXR expression (400×). (E) Quantification showed that the percentage of cases with more than 2% PXR-positive nuclei was significantly higher in EAC than in BE (*P *< 0.01).

To test if the presence of PXR mRNA corresponded with the expression of PXR protein, esophageal biopsies of 39 patients were stained for PXR by immunohistochemistry. Figure 2 depicts representative stainings of PXR on esophageal biopsy specimens of healthy controls, and RE, BE, and adenocarcinoma patients. None of the normal squamous esophageal samples (n = 3) stained positive for PXR (Figure 2A). Also, no specific PXR signal was detected in RE samples (Figure 2B). In patients with histologically confirmed BE (n = 28), six cases of nuclear positivity were found (Figure 2C). In 17/19 adenocarcinoma patients, PXR expression was observed in the nuclei of cancer cells (Figure 2D). This was significantly higher compared to nuclear PXR expression in BE tissue (*P *< 0.01, Figure 2E).

### Exposure to bile acids does not affect PXR mRNA levels, but does induce nuclear translocation

**Figure 3:**
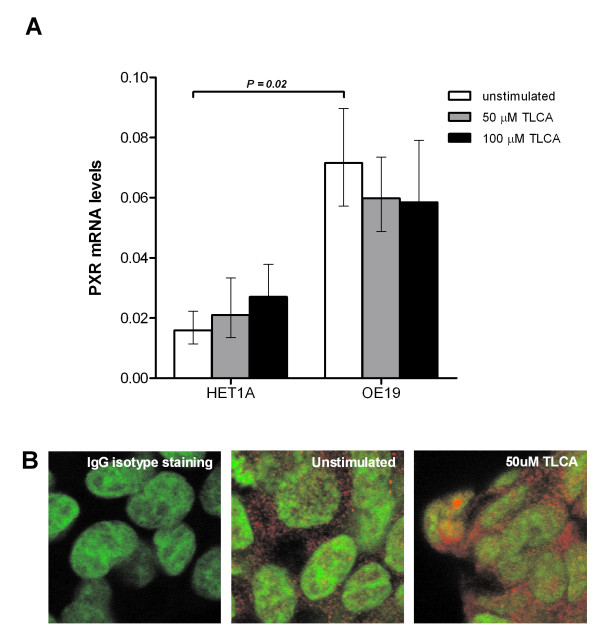
**PXR mRNA levels and nuclear translocation of PXR protein in esophageal cell lines stimulated with bile acids**. (A) PXR mRNA levels are significantly higher in OE19 than in HET1A (*P *= 0.02). Bile stimulation with 50 μM or 100 μM of TLCA did not affect PXR mRNA levels compared to unstimulated conditions. (B) After immunofluorescent staining of PXR (red) and nuclei (green), localization in OE19 cells was visualized by a confocal laser microscope (1000×). In unstimulated cells, PXR was predominantly found in the cytoplasm. Upon 24 h of stimulation with 50 μM of TLCA, PXR translocation from the cytoplasm to the nuclei was observed.

PXR mRNA levels were analyzed in HET1A and OE19 cells upon stimulation with 50 or 100 μM TLCA. PXR levels in the OE19 adenocarcinoma cell line were higher than in the squamous epithelial HET1A cells (*P *= 0.02), but mRNA levels did not differ between unstimulated cells and cells stimulated with TLCA (Figure 3A). Figure 3B shows immunofluorescence of PXR in the nuclei of OE19 cells that were unstimulated, or stimulated with TLCA. Induction with rifampicine was taken as a positive control. More nuclear PXR staining was observed in cells stimulated with 10 μM rifampicine (data not shown) and 50 μM TLCA compared to unstimulated cells, with most intense staining observed in TLCA stimulated OE19 cells. In summary, exposure of adenocarcinoma cells to bile acids and xenobiotics appears to induce nuclear translocation of PXR independent of its gene levels.

### PXR polymorphism 7635AG is associated with BE

**Figure 4:**
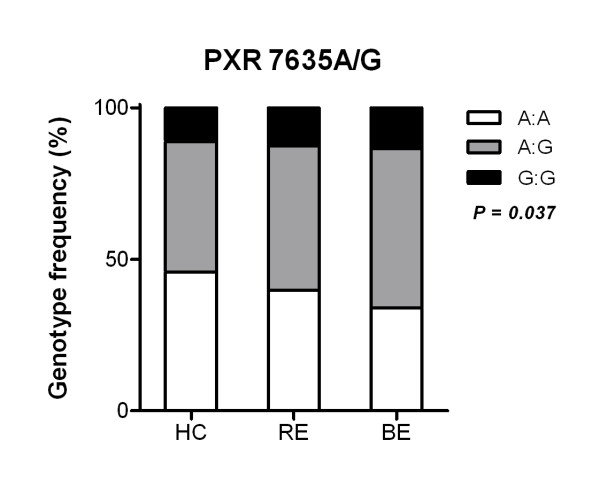
**Genotype distributions of PXR polymorphisms across populations of healthy controls (HC), patients with reflux esophagitis (RE) and Barrett's esophagus (BE)**. The distribution of AA (white), AG (gray) and GG (black) at locus 7635 of the PXR gene are depicted per patient group. As the pathologic condition of the esophagus progresses from healthy to RE to BE, prevalence of ancestral homozygous genotype decreases (*P *= 0.037).

Polymorphisms at location 7635 and 8055 of the PXR gene have previously been found to be located in different linkage disequillibrium blocks and are thought to have an effect on PXR activity [14]. In our cohort the PXR gene polymorphisms were in Hardy-Weinberg Equilibrium. No significant association of SNP -25385C/T with BE or RE was found (*P *> 0.5; data not shown). Allele frequencies of SNP 7635A/G and 8055C/T for patient and healthy control populations are listed in Table 3. Minor allele frequencies of these SNPs were in consensus with previous observations in European control cohorts [14,24,25]. Subjects carrying the SNP 7635G allele had an increased risk of BE (OR 1.36, 95% CI 1.03-1.79). In comparing genotype distributions, an increase was demonstrated in the minor allele frequency among BE patients as compared with RE patients and healthy controls for both 7635A/G and 8055C/T. For SNP 7635A/G this trend was statistically significant (*P *= 0.037, Figure 4).

**Table 3:** Allele frequencies of PXR SNPs at locus 7635 and 8055

SNP		Allele frequency, no. (fraction)			HC *vs *RE	HC *vs *BE
				
locus	Allele	HC	RE	BE	OR (95% CI)	OR (95% CI)
7635	A G	267 (0.674) 129 (0.326)	294 (0.636) 168 (0.364)	298 (0.603) 196 (0.397)	1.18 (0.89-1.57)	***1.36 (1.03-1.79)***
8055	C T	321 (0.863) 51 (0.137)	381 (0.832) 77 (0.168)	397 (0.814) 91 (0.186)	1.27 (0.87-1.87)	1.44 (0.99-2.10)

HC: healthy controls, RE: reflux esophagitis, BE: Barrett's esophagus

## Discussion

The precise pathophysiological mechanisms causing BE is still unclear, but the combination of gastric acid and bile acids from the gastroduodenal reflux is commonly acknowledged as the key factor in the development of BE [26]. At low pH, bile acids are thought to cause esophageal mucosal injury, which has been substantiated both *in vitro *and in animal model systems [27-29].

The NR1I family of orphan nuclear receptors are known to prevent toxic accumulations of xenobiotics within cells by regulating a broad range of cellular transporters [17,30,31]. The nuclear receptor PXR is a member of this family and functions in the enterohepatic organs as a detoxifier and regulator of bile acid homeostasis [12-16]. It can bind a variety of bile acids [32,33] and subsequently regulate the expression of a multitude proteins that transport bile acids across cell membranes [34-36]. These include the multidrug resistance (MDR)1 gene [37,38], which encodes the efflux protein P-glycoprotein that removes xenobiotics from cells [39]. Other bile acid transporters that are induced by PXR include the multidrug resistance associated protein (MRP)2 and 3 [40-42] and the organic anion transporting polypeptide (OATP)1 and 2 [42-44]. From studies in mice it was concluded that the function of PXR is of particular importance when bile acid concentrations reach pathophysiologic levels [33,45].

PXR expression is known in healthy liver and intestinal tract, but in cancer it has yet to be explored. Therefore, in this study we investigated the expression and significance of PXR in esophageal pathology. We did not detect PXR in normal squamous epithelium or in the squamous epithelium of RE patients. PXR did however express at both mRNA and protein level in columnar epithelium, and was significantly lower in adjacent squamous esophageal epithelium of the same patient. In samples from adenocarcinoma patients PXR was clearly observed the nucleus. PXR mRNA levels between BE and EAC do not differ, but nuclear PXR protein expression does increase in EAC. Perhaps, this is an effect of difference posttranscriptional modifications between the stages. It could also indicate a translocation from the cytoplasm to the nucleus occuring during progression from BE to EAC, as our studies showed translocation from cytoplasm to the nuclei of adenocarcinoma cells *in vitro *after stimulation with rifampicine or litholic acid. These processes and their significance to PXR function need to be further explored, and a first step in this could be Western blot analysis on subcellular fractions of BE and EAC cells.

Previous studies have suggested that PXR expression in cancer cells can interfere with the metabolism and responsiveness to chemotherapeutics, such as irinotecon and tamoxifen [46,47]. They suggest this drug resistance involves the metabolizing enzyme CYP3A4, one of the key target genes of PXR [15]. These effects on the metabolism of anticancer agents are especially important considering that PXR ligands include endogenous steroids and bile acids, as well as numerous environmental chemicals and dietary constituents. It has yet to be investigated whether higher levels of PXR in the esophagus also affects responsiveness to chemotherapy.

Given the relatively low rare allele frequency for SNP 8055C/T, our population size may have been insufficient to detect a statistically significant association. Validation of our findings will require a well-characterized population from a multicenter study. Recent studies associate PXR polymorphisms with other pathogenic conditions of the gastrointestinal tract, such as inflammatory bowel disease [20] and primary sclerosing cholangitis [21]. Since associations with the two PXR SNPs in this study are in line with previous findings in IBD [20], this draws attention on a possible link of the functional effect of these SNPs with chronic inflammation. It is well known that inflammation, through the activation of NF-κB pathway leads to a decrease of CAR, PXR and RXR-alpha expression and the expression of their target genes. In addition, it has recently been shown that the mutual repression between PXR and NF-κB signalling pathways provides a molecular mechanism linking xenobiotic metabolism and inflammation [48].

Although it cannot be ruled out that the observed link between BE and PXR levels is not the cause but only the consequence of the metaplasia from squamous to intestinal-type mucosa, the link with PXR-activity associated SNPs suggest a active role of PXR in BE pathophysiology. Further research should focus on the biologic function of PXR in BE and EAC, especially because PXR protein expression was observed in only few nuclei in Barrett's epithelium whereas EAC tissue was abundant with PXR positive nuclei. Here, we chose LCA to study nuclear translocation as it is the endogenous ligand with the highest binding affinity for PXR. As supraphysiological levels of LCA were used to stimulate esophageal cells, further research will be required using extensive stimulation assays that mimick the *in vivo *situation by long-term repetitive stimulations with a mix of bile acids in physiologic concentrations as recently performed [49]. For a complex disease such as BE, development and validation of representative animal models will be of great value to investigate whether PXR plays a protective role in the development of BE or if it has a detrimental effect on neoplastic progression.

## Conclusions

In summary, PXR which is normally not present in the squamous esophageal epithelium, is expressed highly in the columnar esophageal epithelium of BE patients and tumor tissue of EAC patients. At a protein level, this expression appears to be more nuclear in EAC than in BE. Upon stimulation with lithocholic acid, PXR translocates to the nuclei of OE19 adenocarcinoma cells. Together with the observed association of a PXR-activity associated SNPs and BE, this data implies that PXR may have a function in predicting progression and treatment of esophageal disease, though further studies are warranted to support this hypothesis.

## Declaration of competing interest

The authors declare that they have no competing interests.

## Authors' contributions

AW carried out the cell and immunohistochemical studies, participated in all analysis and drafted the manuscript. VM participated in design and acquisition of molecular genetic studies. AC participated in the cell and immunohistochemical studies and contributed to interpretation of data. LM made substantial contributions to acquisition, analysis and interpretation of genetic studies. RP carried out the molecular genetic studies and performed the statistical analysis. HD acted as expert pathologist in analysing and interpreting analysis. PS conceived of the study, and participated in its design and coordination. JK conceived of the study, and participated in its design and coordination. LL participated in study design and coordination and made substantial contributions to interpretation of data and drafting and revising the manuscript. EK participated in study design and made substantial contributions to interpretation of data and was involved in drafting the manuscript and revising it critically for important intellectual content. All authors read and approved the final manuscript

## Pre-publication history

The pre-publication history for this paper can be accessed here:

http://www.biomedcentral.com/1471-230X/11/108/prepub

## References

[B1] Falk GW Barrett's esophagus Gastroenterology 2002 122 1569 1591 10.1053/gast.2002.33427 12016424

[B2] Winters C Spurling TJ Chobanian SJ Curtis DJ Esposito RL Hacker JF Johnson DA Cruess DF Cotelingam JD Gurney MS Barrett's esophagus. A prevalent, occult complication of gastroesophageal reflux disease Gastroenterology 1987 92 118 124 3781178

[B3] Lagergren J Bergstrom R Lindgren A Nyren O Symptomatic gastroesophageal reflux as a risk factor for esophageal adenocarcinoma N Engl J Med 1999 340 825 831 10.1056/NEJM199903183401101 10080844

[B4] Fass R Sampliner RE Malagon IB Hayden CW Camargo L Wendel CS Garewal HS Failure of oesophageal acid control in candidates for Barrett's oesophagus reversal on a very high dose of proton pump inhibitor Aliment Pharmacol Ther 2000 14 597 602 10.1046/j.1365-2036.2000.00749.x 10792123

[B5] Jankowski JA Harrison RF Perry I Balkwill F Tselepis C Barrett's metaplasia Lancet 2000 356 2079 2085 10.1016/S0140-6736(00)03411-5 11145505

[B6] Falk GW Reflux disease and Barrett's esophagus Endoscopy 1999 31 9 16 10.1055/s-1999-13643 10082405

[B7] Falk GW Barrett's esophagus-is it bad for your health? Am J Gastroenterol 2005 100 2622 2623 10.1111/j.1572-0241.2005.00342.x 16393210

[B8] van Soest EM Dieleman JP Siersema PD Sturkenboom MC Kuipers EJ Increasing incidence of Barrett's oesophagus in the general population Gut 2005 54 1062 1066 10.1136/gut.2004.063685 PMC1774890 15857935

[B9] Richter JE Importance of bile reflux in Barrett's esophagus Dig Dis 2000 18 208 216 10.1159/000051401 11356992

[B10] Triadafilopoulos G Acid and bile reflux in Barrett's esophagus: a tale of two evils Gastroenterology 2001 121 1502 1506 10.1053/gast.2001.30090 11729130

[B11] di Pietro M Peters CJ Fitzgerald RC Clinical puzzle: Barrett's oesophagus Dis Model Mech 2008 1 26 31 10.1242/dmm.000273 PMC2561971 19048049

[B12] Zollner G Marschall HU Wagner M Trauner M Role of nuclear receptors in the adaptive response to bile acids and cholestasis: pathogenetic and therapeutic considerations Mol Pharm 2006 3 231 251 10.1021/mp060010s 16749856

[B13] Kliewer SA Moore JT Wade L Staudinger JL Watson MA Jones SA McKee DD Oliver BB Willson TM Zetterstrom RH An orphan nuclear receptor activated by pregnanes defines a novel steroid signaling pathway Cell 1998 92 73 82 10.1016/s0092-8674(00)80900-9 9489701

[B14] Zhang J Kuehl P Green ED Touchman JW Watkins PB Daly A Hall SD Maurel P Relling M Brimer C The human pregnane × receptor: genomic structure and identification and functional characterization of natural allelic variants Pharmacogenetics 2001 11 555 572 10.1097/00008571-200110000-00003 11668216

[B15] Lehmann JM McKee DD Watson MA Willson TM Moore JT Kliewer SA The human orphan nuclear receptor PXR is activated by compounds that regulate CYP3A4 gene expression and cause drug interactions J Clin Invest 1998 102 1016 1023 10.1172/JCI3703 PMC508967 9727070

[B16] Bertilsson G Heidrich J Svensson K Asman M Jendeberg L Sydow-Backman M Ohlsson R Postlind H Blomquist P Berkenstam A Identification of a human nuclear receptor defines a new signaling pathway for CYP3A induction Proc Natl Acad Sci USA 1998 95 12208 12213 10.1073/pnas.95.21.12208 PMC22810 9770465

[B17] Kliewer SA Goodwin B Willson TM The nuclear pregnane × receptor: a key regulator of xenobiotic metabolism Endocr Rev 2002 23 687 702 10.1210/er.2001-0038 12372848

[B18] Krasowski MD Yasuda K Hagey LR Schuetz EG Evolutionary selection across the nuclear hormone receptor superfamily with a focus on the NR1I subfamily (vitamin D, pregnane X, and constitutive androstane receptors) Nucl Recept 2005 3 2 10.1186/1478-1336-3-2 PMC1262763 16197547

[B19] Blumberg B Sabbagh W Juguilon H Bolado J van Meter CM Ong ES Evans RM SXR, a novel steroid and xenobiotic-sensing nuclear receptor Genes Dev 1998 12 3195 3205 10.1101/gad.12.20.3195 PMC317212 9784494

[B20] Dring MM Goulding CA Trimble VI Keegan D Ryan AW Brophy KM Smyth CM Keeling PW O'Donoghue D O'Sullivan M The pregnane × receptor locus is associated with susceptibility to inflammatory bowel disease Gastroenterology 2006 130 341 348 quiz 592 10.1053/j.gastro.2005.12.008 16472590

[B21] Karlsen TH Lie BA Frey Froslie K Thorsby E Broome U Schrumpf E Boberg KM Polymorphisms in the steroid and xenobiotic receptor gene influence survival in primary sclerosing cholangitis Gastroenterology 2006 131 781 787 10.1053/j.gastro.2006.05.057 16952547

[B22] Moons LM Kuipers EJ Rygiel AM Groothuismink AZ Geldof H Bode WA Krishnadath KK Bergman JJ van Vliet AH Siersema PD COX-2 CA-haplotype is a risk factor for the development of esophageal adenocarcinoma Am J Gastroenterol 2007 102 2373 2379 10.1111/j.1572-0241.2007.01373.x 17581270

[B23] Livak KJ Schmittgen TD Analysis of relative gene expression data using real-time quantitative PCR and the 2(-Delta Delta C(T)) Method Methods 2001 25 402 408 10.1006/meth.2001.1262 11846609

[B24] Hustert E Zibat A Presecan-Siedel E Eiselt R Mueller R Fuss C Brehm I Brinkmann U Eichelbaum M Wojnowski L Natural protein variants of pregnane × receptor with altered transactivation activity toward CYP3A4 Drug Metab Dispos 2001 29 1454 1459 11602521

[B25] Bosch TM Deenen M Pruntel R Smits PH Schellens JH Beijnen JH Meijerman I Screening for polymorphisms in the PXR gene in a Dutch population Eur J Clin Pharmacol 2006 62 395 399 10.1007/s00228-006-0108-0 16568343

[B26] Theisen J Peters JH Stein HJ Experimental evidence for mutagenic potential of duodenogastric juice on Barrett's esophagus World J Surg 2003 27 1018 1020 10.1007/s00268-003-7055-z 14560365

[B27] Dvorak K Payne CM Chavarria M Ramsey L Dvorakova B Bernstein H Holubec H Sampliner RE Guy N Condon A Bile acids in combination with low pH induce oxidative stress and oxidative DNA damage: relevance to the pathogenesis of Barrett's oesophagus Gut 2007 56 763 771 10.1136/gut.2006.103697 PMC1954874 17145738

[B28] Harmon JW Johnson LF Maydonovitch CL Effects of acid and bile salts on the rabbit esophageal mucosa Dig Dis Sci 1981 26 65 72 10.1007/BF01307977 7460707

[B29] Sital RR Kusters JG De Rooij FW Kuipers EJ Siersema PD Bile acids and Barrett's oesophagus: a sine qua non or coincidence? Scand J Gastroenterol Suppl 2006 11 17 10.1080/00365520600664219 16782617

[B30] Urquhart BL Tirona RG Kim RB Nuclear receptors and the regulation of drug-metabolizing enzymes and drug transporters: implications for interindividual variability in response to drugs J Clin Pharmacol 2007 47 566 578 10.1177/0091270007299930 17442683

[B31] Kullak-Ublick GA Stieger B Meier PJ Enterohepatic bile salt transporters in normal physiology and liver disease Gastroenterology 2004 126 322 342 10.1053/j.gastro.2003.06.005 14699511

[B32] Staudinger JL Goodwin B Jones SA Hawkins-Brown D MacKenzie KI LaTour A Liu Y Klaassen CD Brown KK Reinhard J The nuclear receptor PXR is a lithocholic acid sensor that protects against liver toxicity Proc Natl Acad Sci USA 2001 98 3369 3374 10.1073/pnas.051551698 PMC30660 11248085

[B33] Schuetz E Strom S Promiscuous regulator of xenobiotic removal Nat Med 2001 7 536 537 10.1038/87856 11329050

[B34] Kullak-Ublick GA ABC transporter regulation by bile acids: where PXR meets FXR J Hepatol 2003 39 628 630 10.1016/s0168-8278(03)00397-0 12971975

[B35] Matic M Mahns A Tsoli M Corradin A Polly P Robertson GR Pregnane × receptor: promiscuous regulator of detoxification pathways Int J Biochem Cell Biol 2007 39 478 483 10.1016/j.biocel.2006.08.017 17188925

[B36] Schinkel AH Jonker JW Mammalian drug efflux transporters of the ATP binding cassette (ABC) family: an overview Adv Drug Deliv Rev 2003 55 3 29 10.1016/s0169-409x(02)00169-2 12535572

[B37] Geick A Eichelbaum M Burk O Nuclear receptor response elements mediate induction of intestinal MDR1 by rifampin J Biol Chem 2001 276 14581 14587 10.1074/jbc.M010173200 11297522

[B38] Synold TW Dussault I Forman BM The orphan nuclear receptor SXR coordinately regulates drug metabolism and efflux Nat Med 2001 7 584 590 10.1038/87912 11329060

[B39] Ambudkar SV Dey S Hrycyna CA Ramachandra M Pastan I Gottesman MM Biochemical, cellular, and pharmacological aspects of the multidrug transporter Annu Rev Pharmacol Toxicol 1999 39 361 398 10.1146/annurev.pharmtox.39.1.361 10331089

[B40] Kast HR Goodwin B Tarr PT Jones SA Anisfeld AM Stoltz CM Tontonoz P Kliewer S Willson TM Edwards PA Regulation of multidrug resistance-associated protein 2 (ABCC2) by the nuclear receptors pregnane × receptor, farnesoid X-activated receptor, and constitutive androstane receptor J Biol Chem 2002 277 2908 2915 10.1074/jbc.M109326200 11706036

[B41] Borst P Zelcer N van de Wetering K MRP2 and 3 in health and disease Cancer Lett 2006 234 51 61 10.1016/j.canlet.2005.05.051 16387425

[B42] Rosenfeld JM Vargas R Xie W Evans RM Genetic profiling defines the xenobiotic gene network controlled by the nuclear receptor pregnane × receptor Mol Endocrinol 2003 17 1268 1282 10.1210/me.2002-0421 12663745

[B43] Miki Y Suzuki T Kitada K Yabuki N Shibuya R Moriya T Ishida T Ohuchi N Blumberg B Sasano H Expression of the steroid and xenobiotic receptor and its possible target gene, organic anion transporting polypeptide-A, in human breast carcinoma Cancer Res 2006 66 535 542 10.1158/0008-5472.CAN-05-1070 16397270

[B44] Glaeser H Bailey DG Dresser GK Gregor JC Schwarz UI McGrath JS Jolicoeur E Lee W Leake BF Tirona RG Intestinal drug transporter expression and the impact of grapefruit juice in humans Clin Pharmacol Ther 2007 81 362 370 10.1038/sj.clpt.6100056 17215845

[B45] Xie W Radominska-Pandya A Shi Y Simon CM Nelson MC Ong ES Waxman DJ Evans RM An essential role for nuclear receptors SXR/PXR in detoxification of cholestatic bile acids Proc Natl Acad Sci USA 2001 98 3375 3380 10.1073/pnas.051014398 PMC30661 11248086

[B46] Raynal C Pascussi JM Leguelinel G Breuker C Kantar J Lallemant B Poujol S Bonnans C Joubert D Hollande FJ Pregnane × Receptor (PXR) expression in colorectal cancer cells restricts irinotecan chemosensitivity through enhanced SN-38 glucuronidation Molecular Cancer 2010 9 46 10.1186/1476-4598-9-46 PMC2838814 20196838

[B47] Sane RS Buckley DJ Buckley AR Nalani SC Desai PB Role of human Pregnane × Receptor in tamoxifen and 4-hydroxytamoxifen mediated CYP3A4 induction in primary human hepatocytes and LS174T cells Drug Metab Dispos 2008 36 946 954 10.1124/dmd.107.018598 18299335

[B48] Pascussi JM Vilarem MJ Molecular mechanisms linking xenobiotic metabolism and inflammation Med Sci (Paris) 2008 24 301 305 10.1051/medsci/2008243301 18334180

[B49] Goldman A Chen HD Roesly HB Hill KA Tome ME Dvorak B Bernstein H Dvorak K Characterization of squamous esophageal cells resistant to bile acids at acidic pH: implication for Barrett's esophagus pathogenesis Am J Physiol Gastrointest Liver Physiol 2011 300 G292 302 10.1152/ajpgi.00461.2010 PMC3043651 21127259

